# Renal blood flow and oxygenation

**DOI:** 10.1007/s00424-022-02690-y

**Published:** 2022-04-19

**Authors:** Aurelie Edwards, Vartan Kurtcuoglu

**Affiliations:** 1 Department of Biomedical Engineering, Boston University, 44 Cummington Mall, Boston, MA 02215 USA; 2 Institute of Physiology, University of Zurich, Winterthurerstrasse 190, 8057 Zurich, Switzerland; 3 National Center of Competence in Research, Kidney.CH, University of Zurich, Zurich, Switzerland; 4 Zurich Center for Integrative Human Physiology, University of Zurich, Zurich, Switzerland

**Keywords:** Kidney, Perfusion, Autoregulation, Oxygenation

## Abstract

Our kidneys receive about one-fifth of the cardiac output at rest and have a low oxygen extraction ratio, but may sustain, under some conditions, hypoxic injuries that might lead to chronic kidney disease. This is due to large regional variations in renal blood flow and oxygenation, which are the prerequisite for some and the consequence of other kidney functions. The concurrent operation of these functions is reliant on a multitude of neuro-hormonal signaling cascades and feedback loops that also include the regulation of renal blood flow and tissue oxygenation. Starting with open questions on regulatory processes and disease mechanisms, we review herein the literature on renal blood flow and oxygenation. We assess the current understanding of renal blood flow regulation, reasons for disparities in oxygen delivery and consumption, and the consequences of disbalance between O_2_ delivery, consumption, and removal. We further consider methods for measuring and computing blood velocity, flow rate, oxygen partial pressure, and related parameters and point out how limitations of these methods constitute important hurdles in this area of research. We conclude that to obtain an integrated understanding of the relation between renal function and renal blood flow and oxygenation, combined experimental and computational modeling studies will be needed.

## Introduction

The kidneys fulfill a multitude of functions that can be described, in very general terms, as blood conditioning. To this end, mammalian kidneys receive approximately 20% of the cardiac output under resting conditions, which is more than they need to meet their own metabolic demand, as evinced by the low oxygen extraction ratio of 10–15%. At the same time, there is marked heterogeneity of both blood perfusion and oxygen consumption in renal tissues, which renders parts of the kidney susceptible to hypoxia despite overall excess of oxygen. While the partial pressure of oxygen (pO_2_) in renal cortical tissue is in the range of 20–60 mmHg, the outer and inner medulla show characteristic pO_2_ values of 15–30 mmHg and < 15 mmHg, respectively [1]. Such regional variations in pO_2_ are owed, in part, to the renal vascular architecture.

As interlobular arteries ascend from the cortico-medullary junction to the renal capsule, they supply the afferent arterioles of juxtamedullary glomeruli at lower levels and of cortical glomeruli at higher levels. Thus, the cortical and medullary vasculatures are connected in series. The efferent arterioles of superficial glomeruli give rise to the peritubular capillaries that surround proximal and distal tubules in the cortex, while the efferent arterioles of juxtamedullary glomeruli give rise to descending vasa recta that supply the medulla, and that turn back at varying depths to form ascending vasa recta. These observations already imply that blood flow regulation in the kidney is more complex than in organs where metabolic demand is the primary determinant. Exceeding regulatory limits or dysfunction of renal blood flow (RBF) regulation can lead to renal ischemia, which is an important factor in both the genesis of acute kidney injury and the development of chronic kidney disease. To investigate the mechanisms of renal pathogenesis, and for clinical decision-making, understanding the determinants of RBF and renal tissue oxygenation is required. However, despite recent advances, obtaining an integrated picture remains challenging.

The ability of the kidney to perform multiple functions simultaneously is sustained by a large number of neuro-hormonal agents that interact in numerous ways, with signaling cascades and feedback loops that are difficult to disentangle. Many questions, ranging from fundamental regulatory processes to specific disease mechanisms, have yet to be resolved: To what extent are cortical and medullary oxygenation independently regulated? What is the role of spatial oxygen gradients in renal tissue in the regulation of erythropoiesis? What are the relative contributions of vasa recta and juxtamedullary arterioles to the control of medullary blood flow (MBF) in vivo? What is the role of MBF in pressure natriuresis? What role do changes in regional hemodynamics and tissue oxygenation play in renal disease and kidney injury? How do age and sex influence the regulation of RBF and tissue oxygenation?

In the following, we review the current literature on RBF and oxygenation beginning with the regulation of blood flow. We consider reasons for disparities between oxygen delivery and consumption, as well as the consequences of the disbalance between O_2_ delivery, consumption, and removal. Finally, we describe present hurdles in studying renal blood flow and oxygenation and close the circle by showing how the open questions listed above are linked to limitations of available methods for measuring and computing blood flow rate, velocity, renal tissue oxygenation, and related parameters.

## Renal blood flow

Renal blood flow or renal blood flow rate may refer both to flow through the entire organ, or through parts thereof. At the highest level, cortical and medullary blood flow needs to be differentiated. Total RBF is maintained approximately constant over a wide pressure range, e.g., from about 100 to 160 mmHg in rats [2, 3]. Current understanding is that the medulla receives approximately 10% of the total RBF [4, 5] through juxtamedullary efferent arterioles that give rise to vasa recta, which are surrounded by pericytes that allow for independent control of medullary blood flow by vasoactive substances. Since MBF is small compared to total renal blood flow, moderate changes in MBF may not have a measurable effect on total RBF. The autoregulation of RBF is achieved by intrinsic mechanisms that modulate the resistance of (mostly) preglomerular vessels, and is thereby coupled to the autoregulation of the glomerular filtration rate (GFR). RBF is also regulated by extrinsic mechanisms, some of which differentially alter the resistance of afferent versus efferent arterioles, allowing for the partial decoupling of GFR and RBF, and/or differentially impact the cortical versus medullary circulation. While RBF autoregulation affects renal oxygenation, control of O_2_ delivery is not its primary role.

### Intrinsic mechanisms of renal blood flow regulation

The autoregulation of RBF is mediated primarily by two mechanisms, the fast myogenic response and the slower macula densa (MD) tubulo-glomerular feedback (TGF). Two other autoregulatory mechanisms have been proposed, one that operates at a low frequency [6] and one that involves crosstalk between the connecting tubule and the afferent arteriole [7], but their contributions appear smaller and they remain poorly understood [8]. Modeling studies have significantly expanded our understanding of these intrinsic regulatory mechanisms, including their relative contributions and interactions and how they are affected by specific pathological conditions [9, 10].

#### Myogenic mechanism

Preglomerular vessels constrict when subjected to an increase in transmural pressure difference, a response known as the myogenic mechanism; the resulting increase in vascular resistance lowers perfusion. Thus, the myogenic response acts to stabilize RBF and GFR and to protect the glomerulus from increases in systolic blood pressure.

The myogenic response is initiated by mechano-receptors that transduce the mechanical signal into transmembrane currents, which then activate cytosolic signaling cascades that amplify the signal and ultimately cause smooth muscle cell contraction. The first step, i.e., mechanical-electrical transduction, may involve cell surface integrin receptors [11], mechanosensitive ion channels such as ENaC/degenerins [12], transient receptor potential channels, and stretch-activated G-protein coupled receptors [13]. These pathways, the relative contributions of which remain to be fully elucidated, depolarize the membrane, thereby activating voltage-gated Ca^2+^ channels and triggering Ca^2+^ influx. The resulting increase in cytosolic Ca^2+^ triggers the release of Ca^2+^ from sarcoplasmic reticulum stores, all of which stimulate myosin light chain kinase and inhibit myosin light chain phosphatase, thus eliciting cross-bridge formation and muscle contraction.

#### Tubulo-glomerular feedback

TGF modulates the resistance of the afferent arteriole in response to changes in NaCl delivery to the macula densa, a small cluster of tubular epithelial cells between the cortical thick ascending limb and the distal convoluted tubule. Specifically, an increase in tubular NaCl concentration at the MD, reflecting a higher rate of NaCl filtration and/or reduced reabsorption in pre-MD segments, triggers contraction of the afferent arteriole, thus reducing blood flow and the filtration rate. Conversely, a decrease in NaCl concentration at the MD leads to dilation of the arteriole.

The mechanisms underlying TGF are not completely understood. Elevations in NaCl delivery to the MD stimulate the apical uptake of Na^+^, K^+^, and Cl^−^ via the Na-K-2Cl cotransporter NKCC2, thereby raising intracellular Cl^−^ levels and altering the basolateral membrane potential of MD cells. One or both of these signals activate the release of ATP and/or its breakdown product adenosine into extra-glomerular mesangial cells and adjacent vascular smooth muscle cells (VSMCs) on the afferent arteriole. The binding of ATP and adenosine to, respectively, purinergic P2X and adenosine A1 receptors on VSMCs triggers signaling cascades that raise intracellular Ca^2+^ levels, thereby activating contraction and reducing the diameter of the arteriole. The subsequent reduction in the filtration rate in turn reduces NaCl delivery to the MD, thus closing the negative feedback loop. Note that MD signaling may also involve vasoactive factors, including prostaglandins and nitric oxide [13].

Changes in NaCl delivery to the MD may result from variations in the filtration pressure and/or in proximal reabsorption. Thus, TGF acts not only to regulate glomerular pressure and flow, but also stabilizes fluid and electrolyte delivery to the distal nephron, which facilitates the hormone-mediated fine-tuning of reabsorption and secretion therein [14]. TGF may be modulated by the connecting tubule-glomerular feedback, a positive feedback mechanism that dilates the afferent arteriole when high concentrations of sodium are detected in the connecting tubule, thereby increasing filtration [15]. The connecting tubule-glomerular feedback is abolished when the epithelial channel ENaC is inhibited, and there is evidence that it contributes to TGF resetting during high salt intake [16].

### Extrinsic mechanisms of renal blood flow regulation

RBF is also regulated by systemic and paracrine factors. Cortical versus medullary differences in vascular structure, receptor expression, and hormone synthesis rates allow for the differential regulation of cortical (CBF) versus medullary blood flow and may help to prevent medullary hypoxia even when RBF is reduced [1].

#### Hormonal signals

Renal vascular tone is controlled by many vasoconstrictors (including angiotensin II, endothelins [17], norepinephrine, vasopressin [18], and reactive oxygen species [19]) and vasodilators (including nitric oxide, adenosine, prostaglandins [20], and bradykinin [21]**).** Many of these factors also affect tubular function, i.e., they modulate both O_2_ supply and O_2_ demand (see below). Of particular importance are angiotensin II (Ang II), nitric oxide (NO), and adenosine.

Ang II is the most potent effector of the renin-angiotensin-aldosterone system. Its effects are mediated by two receptors, AT_1_ (predominantly expressed) and AT_2_. The binding of Ang II to AT_1_ elicits vasoconstriction and stimulates tubular sodium reabsorption, whereas binding to AT_2_ causes vasodilation and favors natriuresis. In the renal vasculature, Ang II (via AT_1_) augments the resistance of efferent arterioles more than that of afferent arterioles, thereby decreasing RBF while maintaining GFR [22].

While Ang II reduces CBF [23], it can induce a paradoxical increase in MBF owing to its indirect effects. As reviewed by Evans et al. [24], Ang II directly constricts medullary vessels but it also activates the release of vasodilator paracrine factors (such as prostaglandins and NO) from vascular, tubular, and interstitial cells. The net impact of Ang II on medullary blood flow depends on the relative levels of these effects, as well as the source of Ang II (systemic versus intra-renal).

NO is a vasodilator that is produced by both tubular and vascular cells at significantly higher rates in the medulla than in the cortex [25]. NO synthase inhibition studies have shown that NO plays an important role in maintaining MBF and medullary O_2_ supply under both acute and chronic conditions [26]. Subpressor doses of Ang II stimulate tubular NO synthesis, and the subsequent diffusion of NO towards adjacent pericytes, referred to as tubulo-vascular crosstalk, promotes vasodilation and thereby buffers the vasoconstrictor actions of Ang II [27].

Adenosine is a breakdown product of ATP, and, like NO, it is more concentrated in the medulla than in the cortex [28]. Adenosine acts a paracrine signal via two receptors, A_1_AR and A_2_AR [29]. Activation of A_1_AR constricts afferent arterioles and lowers single nephron GFR and is an essential component of TGF (see above). In contrast, activation of A_2_AR dilates postglomerular vessels. While A_1_AR-mediated constriction is the dominant effect of adenosine in superficial nephrons, A_2_AR-mediated vasodilation prevails in juxtamedullary nephrons, where it acts to enhance MBF [28]. Of note, the release of adenosine by cells of the medullary thick ascending limb (mTAL) is transport-dependent and stimulated by hypoxia [30].

While Ang II stimulates the (counteracting) release of NO, Ang II and adenosine cooperate in inducing afferent arteriole contraction [29]. Moreover, other vasoactive agents, such as endothelin-1 and prostaglandin E_2_, contribute to tubulo-vascular crosstalk in the renal medulla [31]. Even though a full description is beyond the scope of this review, it is important to recognize that the hormonal and neural factors that regulate renal perfusion (and tubular transport, see below) do not act independently but instead interact in synergistic and/or antagonistic ways.

#### Neural signals

Renal blood flow is also modulated by renal sympathetic nerve activity. Renal afferent nerves, situated mainly in the pelvis, are activated by mechanical and chemical signals (including Ang II) and strongly regulate central sympathetic outflow [32]. Renal efferent nerves are found predominantly in the nephron segments and vasculature of the renal cortex and outer medulla [33]. They release norepinephrine, which provokes contraction of the afferent and efferent arterioles via α_1_ and α_2_ adrenoreceptors, thereby reducing RBF and GFR. A role for sympathetic nerve-derived ATP in modulating vasa recta diameter and regulating MBF specifically has also been proposed [34].

Studies compiled by Evans et al. [35] suggest that, with some exceptions, vasoconstrictors appear to decrease CBF more than MBF, whereas most vasodilators appear to increase MBF more than CBF. Aside from regional differences in hormone production rates and receptor expression, another possible factor may be that juxtamedullary arterioles are significantly larger than cortical arterioles, so that their resistance is less impacted, in relative terms, by vasoconstrictor-induced reductions in diameter [35].

## Renal oxygenation

The interplay between renal O_2_ delivery and consumption is complex. Oxygen delivery is proportional to total RBF, and thus directly dependent on blood flow regulation. Oxygen consumption is primarily driven by tubular demand, which, in turn, is dependent on GFR. Increase in total RBF leads to a proportional increase in GFR under normal physiologic conditions [36]. Regional variations in O_2_ delivery and consumption produce spatial pO_2_ gradients that are of relevance for the regulation of erythropoietin (EPO) production [37]. At the same time, areas with lower pO_2_ under normal conditions are at increased risk of hypoxic damage when the balance between O_2_ delivery and consumption shifts. We note that the term “O_2_ delivery” can be ambiguous: it can refer to the O_2_ carried by the blood supplying a given renal region, or it can refer to the O_2_ that has exited the blood stream and diffused into tissue. We will use “O_2_ delivery to tissue” to refer to the latter.

### Determinants of regional pO_2_

**Fig. 1:**
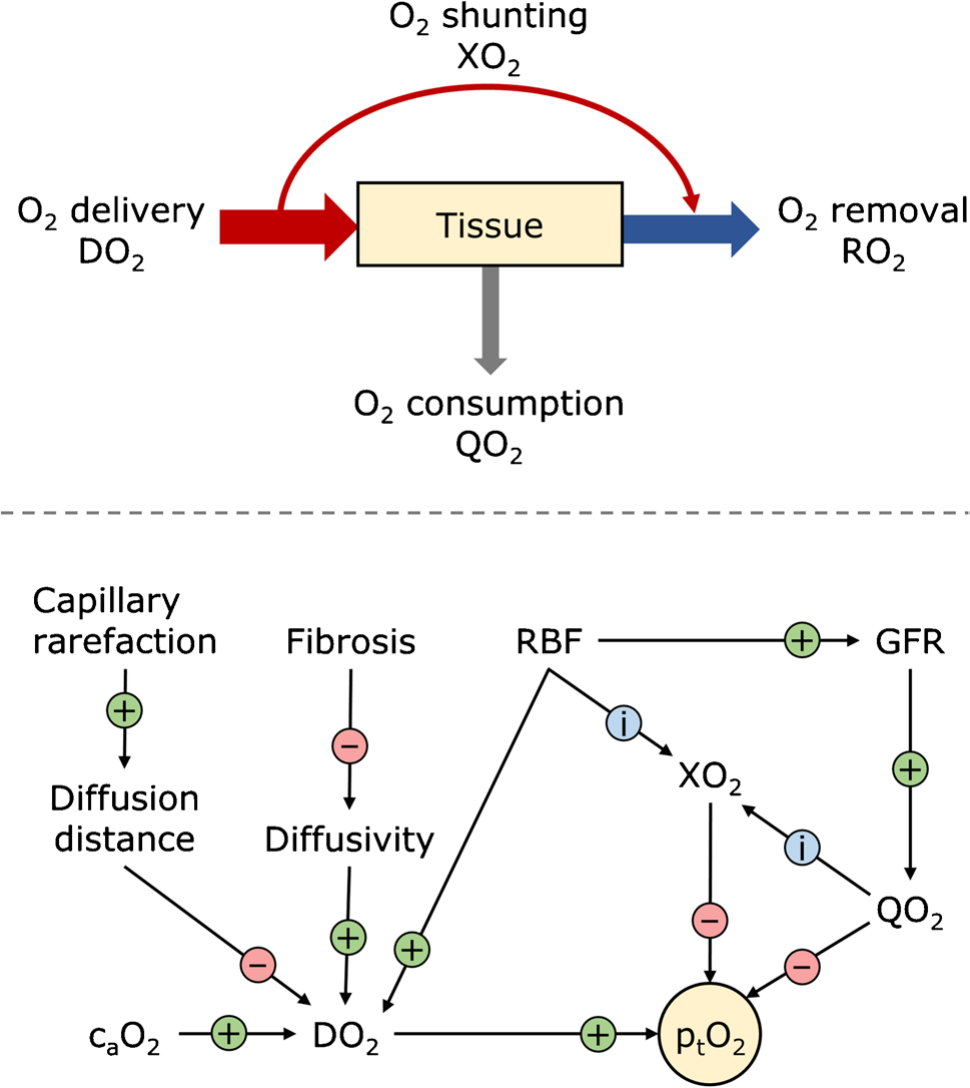
Key factors determining oxygen partial pressure in tissue. Top panel: Tissue oxygenation is a function of O_2_ delivery (DO_2_), consumption (QO_2_), and removal (RO_2_). About 10–15% of O_2_ delivered to the kidney is consumed under normal physiologic conditions. Not all renal tissues are supplied equally, which is, in part, due to arterial-to-venous oxygen shunting (XO_2_). O_2_ not consumed by the kidney is removed by venous efflux. Bottom panel: Tissue partial pressure of oxygen (p_t_O_2_) is dependent on factors that influence O_2_ delivery, consumption, and removal. Circled + and − signs indicate the effect of an increase in the factor upstream of the corresponding arrow on the parameter pointed by the arrowhead under the assumption that everything else remains the same. Circled i indicates that an increase in the respective factor will influence the indicated parameter. Only selected factors are shown. An increase in renal blood flow (RBF) increases DO_2_ and glomerular filtration rate (GFR), and may influence XO_2_. Whether XO_2_ increases is dependent on the location of the tissue under observation, among other factors. XO_2_ is also influenced by QO_2_. Since QO_2_ produces the arterial-to-venous pO_2_ gradients necessary for XO_2_, an increase in consumption will likely, but not necessarily, increase shunting; the local arrangement of O_2_ sinks and sources plays a role as well. More shunting leads to reduced p_t_O_2_. Increased GFR leads to higher QO_2_, as O_2_ demand for Na^+^ reabsorption increases, and thereby to reduced p_t_O_2_. DO_2_ increases with increased arterial blood oxygen concentration (c_a_O_2_) and partial pressure. Capillary rarefaction and fibrosis reduce oxygen delivery to tissue due to increased diffusion distance and reduced diffusivity, respectively

Local pO_2_ is determined by the rate of O_2_ delivery, consumption, and removal, and by local tissue and blood properties (Fig. 1). Increase in O_2_ demand and removal, or decrease in the rate of delivery to tissue, will reduce local pO_2_. The same will occur when the affinity of hemoglobin for O_2_ is increased due to, e.g., alkalosis, or due to pathologic changes in tissue properties such as fibrosis. In the medulla, O_2_ delivery is limited by the requirement for low blood flow through the vasa recta to maintain the osmolality gradients produced by the countercurrent arrangement of descending and ascending vessels and tubules [38]. The process in the kidney with the highest O_2_ demand is synthesis of ATP. Only about 5% of ATP is produced anaerobically [39]. Most of the ATP is utilized to drive tubular Na^+^ reabsorption primarily in the proximal tubule and the thick ascending limb (TAL) of the loop of Henle, where O_2_ consumption is, consequently, high. The efficiency of O_2_ utilization for Na^+^ reabsorption varies between tubular segments. It is higher in the proximal tubule than in the TAL [40]. Also, the distribution of Na^+^ reabsorption is dependent on the level of dietary Na^+^ intake. Increase in GFR leads to higher O_2_ consumption due to increased delivery and consequent higher reabsorption of Na^+^. Elevation of blood glucose levels leads to increased reabsorption of glucose and Na^+^ in the proximal tubule by sodium–glucose cotransporters, and thereby to increased O_2_ consumption [41].

Oxygen is removed by venous outflow. Not all O_2_ travels along the full length of the renal vasculature: O_2_ can be shunted radially from one vessel or vessel segment to another through adjacent tissue, provided that they are within diffusion distance of each other and that there is a driving pO_2_ gradient. This is the case in the vasa recta and in parts of the preglomerular vasculature. As blood flows through the descending vasa recta towards the inner medulla, O_2_ is delivered to the surrounding tissue, where it is, in part, consumed by the nephrons. The remaining O_2_ diffuses into the ascending vasa recta and is transported back in direction outer medulla. This shunting from descending to ascending vasa contributes to medullary hypoxia [42], in particular when reduced perfusion and/or elevated O_2_ consumption increase O_2_ gradients favorable to shunting, thereby further reducing O_2_ delivery to tissue [1]. Complicating matters, blood does not have to follow the full length of the vasa recta, but may take a shortcut from descending to ascending vasa recta through the peritubular capillary plexus. In general, the intricate three-dimensional vascular organization of the outer medulla adds a layer of complexity to the understanding of shunting mechanisms [43–45].

### Regulation mechanisms

**Fig. 2:**
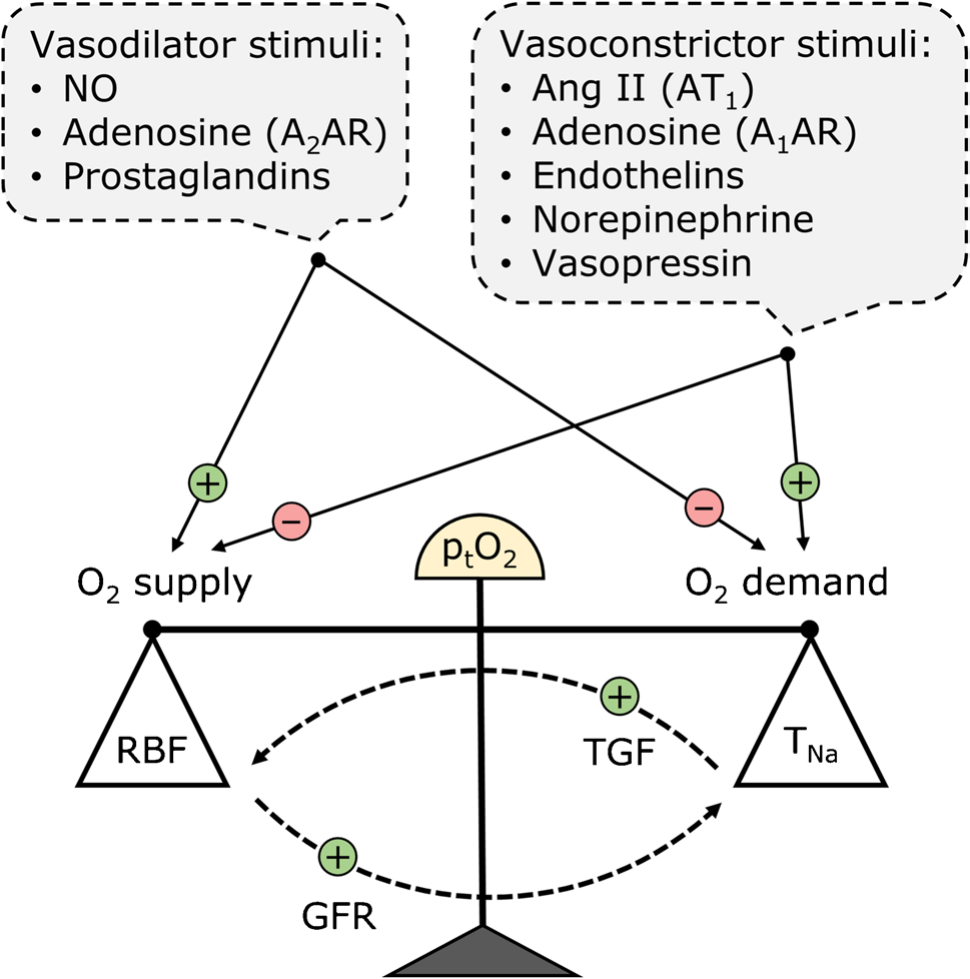
Regulation of tissue pO_2_ (p_t_O_2_) by neuro-hormonal agents. The vasoconstrictor factors that reduce RBF and O_2_ delivery (DO_2_) generally also stimulate sodium reabsorption (T_Na_) and increase O_2_ consumption (QO_2_). Conversely, the vasodilator factors that increase RBF and DO_2_ may also act to reduce T_Na_ and QO_2_. However, these effects are blunted by two mechanisms: increasing RBF also raises GFR and therefore T_Na_, whereas increases in T_Na_ in the proximal tubule and the ascending limb may reduce NaCl delivery to the macula densa and raise RBF via tubulo-glomerular feedback (TGF). Hence, the effectiveness of p_t_O_2_ regulation also depends on how neuro-hormonal agents modulate the coupling between RBF and GFR (i.e., by changing the filtration fraction), or between T_Na_ and QO_2_ (i.e., by changing the metabolic efficiency of Na^+^ transport). These effects are not explicitly shown in the figure (see text). Not shown either are the synergistic and antagonistic effects between various neuro-hormonal agents

In other organs, the end-products of metabolism regulate O_2_ supply without affecting O_2_ consumption. In the kidney, since GFR determines the rate of sodium reabsorption (T_Na_), increases in RBF and GFR raise O_2_ consumption concomitantly with O_2_ supply, a positive feedback loop that is ill-suited to match O_2_ supply and demand (Fig. 2). However, two general mechanisms dissociate O_2_ consumption from O_2_ supply, namely, (i) the decoupling of GFR from RBF via differential modulation of pre- and postglomerular resistance, which alters the filtration fraction and (ii) changes in metabolic efficiency, as reflected by the Na^+^ transport-to-O_2_ consumption ratio (T_Na_/Q_O2_) [46]. This ratio is altered by shifts in T_Na_ to segments with higher/lower passive reabsorption capacity, changes in paracellular permeability, and variations in the efficiency of ATP production in mitochondria. We note that there is little evidence that the kidney purposely regulates T_Na_/Q_O2_ in order to maintain kidney oxygenation when pO_2_ increases or decreases, such as during ischemia [47].

Many of the neuro-humoral factors that regulate blood flow and O_2_ supply also modulate the filtration fraction and T_Na_/Q_O2_, in particular Ang II, NO, and adenosine. As described above, Ang II preferentially constricts efferent arterioles, which raises the filtration fraction and reduces O_2_ supply relative to O_2_ consumption, thereby lowering cortical pO_2_ [23]. Moreover, Ang II stimulates Na^+^ transporter abundance and activity, particularly in the distal nephron [48] where T_Na_/Q_O2_ is lower, which also raises O_2_ consumption. Palm and colleagues recently demonstrated that activation of the renin-angiotensin-aldosterone system by a low Na^+^ diet increased Q_O2_ and lowered cortical pO_2_ but raised medullary pO_2_ [49]; a similar low-salt diet-induced reversal of pO_2_ gradients was previously observed [50]. Of note, AT1 receptor blockade restored pO_2_ without reducing Q_O2_, confirming the major impact of Ang II on renal O_2_ delivery [49].

Conversely, NO acts both to increase MBF and to lower Q_O2_ by inhibiting distal Na^+^ transport and enhancing the efficiency of Na^+^ reabsorption. NO reduces NKCC2 activity in the mTAL [51], and it reduces T_Na_ and vasopressin-stimulated water permeability in the collecting ducts; its effects in the proximal tubule remain controversial [52, 53]. Computational studies suggest that under basal conditions, the NO–induced decrease in Q_O2_ and increase in O_2_ supply contribute to a similar extent to the maintenance of medullary pO_2_ [54]. In addition, NO inhibits respiration in renal tubules [55] by blocking complex IV in mitochondria. Accordingly, NO synthase inhibition reduces renal perfusion and preferentially decreases medullary pO_2_, both acutely and chronically [56].

Adenosine formation is activated by low ATP levels. A_1_AR activation stimulates Na^+^ and fluid reabsorption in the proximal tubule but inhibits T_Na_ in the mTAL, thereby shifting O_2_ consumption towards the cortex where O_2_ is more plentiful [29]. In addition, A_2_AR-mediated enhancement of MBF increases O_2_ supply to the medulla. Thus, similar to NO, adenosine plays an important role in preserving medullary oxygenation [57].

Norepinephrine released by renal efferent nerves also modulates Na^+^ and water reabsorption, as well as intra-renal renin release [33]**.** Thus, renal sympathetic nerve activity likely also affects O_2_ consumption and T_Na_/Q_O2_ in the kidney.

## Impact of dysregulation

A shift in the balance of O_2_ delivery, consumption, and removal can be caused by acute and chronic pathologies, but may also lead to such. While in the healthy kidney all regions receive enough O_2_ to maintain their respective normal physiologic pO_2_, loss of cortical peritubular capillaries may critically reduce O_2_ delivery [58]. Together with the inherent increase in diffusion distances, such capillary rarefaction may cause hypoxia.

Hypoxia has been proposed as a key element in the development and progression of chronic kidney disease (CKD) [59]: Starting from an initial glomerular injury by any mechanism, postglomerular peritubular vascular insufficiency develops, leading to reduced blood flow and reduced O_2_ delivery to tissue. The ensuing hypoxia causes tubular injury and inflammation. This also impacts adjacent capillaries, damaging their respective glomeruli which had previously been unharmed. From there, the cycle repeats. However, conclusive evidence for such a causal link between hypoxia and CKD is lacking [60, 61], even though there are clinical indications for an association between hypoxia and CKD progression [62]. Hypoxia has also been proposed as one of the mechanisms that can lead to acute kidney injury (AKI), which is increasingly recognized as a risk factor for CKD [63]. At least for some forms of AKI, the link to hypoxia appears to be clearer than in the case of CKD: When a decrease in RBF occurs, e.g., due to surgical intervention, O_2_ delivery is temporarily reduced, putting particularly the mTAL with its high O_2_ demand and low medullary blood flow in danger of hypoxic damage [64]. In this case, the S3 segment of the proximal tubule is also at risk, although to a lesser extent. When both O_2_ delivery and transport function temporarily cease without hypothermia, as in warm ischemia reflow animal experiments, the S3 segment is primarily affected [65]. In contrast to the TAL, which can maintain itself by anaerobic glycolysis under such conditions, the proximal tubule is dependent on O_2_ [38].

In hypertension, a shift of Na^+^ reabsorption towards the energetically less efficient TAL appears to contribute, together with other factors, to reduced tissue pO_2_. In diabetes mellitus, O_2_ consumption is increased due to mitochondrial dysfunction, reduced Na^+^ reabsorption efficiency, and glomerular hyperfiltration [66, 67]. In general, conditions that lead to glomerular hyperfiltration have the potential to contribute to hypoxia if dysregulation ensues. An area where the contribution of dysregulation of blood flow and oxygenation is particularly difficult to quantify is the development of anemia in CKD. Renal EPO-producing (REP) cells are specialized tubulo-interstitial cells located, under normal conditions, at the cortico-medullary border where there is a steep spatial gradient in pO_2_. It is hypothesized that REP cells integrate, among other cues, information on O_2_ availability and consumption to regulate EPO [37]. As CKD develops, these cells stop producing EPO and dedifferentiate. However, to what extent changes in the spatial pO_2_ gradient at the cortico-medullary border may contribute remains unclear.

## Hurdles in studying renal blood flow and oxygenation

As in other areas of physiology, kidney research has progressed in tandem with technological innovations. To advance our understanding of renal function, we are dependent on accurate measurements, ideally ones that can be carried out in vivo in a noninvasive fashion to minimize confounding factors. Limitations of available methods for measuring RBF and oxygenation constitute hurdles that must be overcome or bypassed before many of the open questions in this area can be answered. For instance, to understand the role of renal tissue oxygen gradients in the regulation of erythropoiesis, we need to accurately quantify local differences in pO_2_, which is currently not possible [68]. Likewise, to evaluate the role that changes in regional hemodynamics and tissue oxygenation play in renal disease and kidney injury, reliable, quantitative information on RBF and pO_2_ with high spatial resolution is needed. However, shortcomings of measurement methods, including the comparably low resolution of noninvasive approaches, and introduction of local perturbations by invasive techniques, leave us with sparse data.

Most questions are open not only because of limitations in measurement methods, but also due to a lack of tools that would enable the integration of complex data into a bigger picture. For example, studies in rats have shown that medullary pO_2_ may decrease during moderate to severe cortical ischemia even as MBF is maintained [69], possibly as the result of the complex architecture of the renal vasculature [1]. Yet to reliably quantify to what extent the regulation of cortical and medullary oxygenation is independent, a quantitative model is needed; mental representations are not sufficient. Such models could contribute to quantifying the relative contributions of vasa recta and juxtamedullary arterioles to the control of MBF. The use of computational methods in renal physiology is not new but has only spread slowly. For instance, such methods have been employed to investigate the role of MBF in pressure natriuresis, indicating that the modulation of interstitial composition by MBF strongly affects hydration status and the excretion of water, but not that of NaCl [70]. In contrast, observations in rats suggested that changes in medullary perfusion may modulate both sodium and water excretion and thereby alter effective circulating volume [19, 71]. However, recent findings do not support a key role of MBF in regulating blood pressure in humans [72, 73].

In the following sections, we discuss the capabilities and limitations of current methods for measuring and calculating RBF and renal oxygenation, differentiating between invasive, noninvasive, and computational approaches. While blood flow on the whole organ level lends itself well to noninvasive acquisitions, localized high-resolution measurements necessitate invasive methods. Renal blood flow or renal blood flow rate is measured in units of volume per time, e.g., milliliters or liters of blood per minute. In contrast, perfusion is a volume (or mass) averaged metric, representing the volume of blood supplied to a unit volume (or unit mass) of tissue per unit time. Blood velocity gives the direction and speed of blood at a specific location in units of distance traveled per time. When both blood velocity and vessel morphology are known, the blood flow rate can be calculated by spatial integration of the velocity field. The average blood velocity in a vessel is then defined as the blood flow rate through that vessel divided by the vessel’s cross-sectional area.

### Methods for noninvasive measurements

Ultrasound and magnetic resonance imaging (MRI) are used clinically and in animal studies to acquire RBF data. Phase-contrast MRI (PC-MRI) can produce images with controlled sensitivity to flow. In combination with magnitude images to capture anatomy, it allows for the acquisition of spatially resolved blood velocity fields in larger vessels such as the renal artery [74]. While not inherently complicated to use, PC-MRI requires strict adherence to validated protocols for producing reliable results. In contrast to PC-MRI, arterial spin labeling (ASL) MRI can yield data on renal perfusion, i.e., distribution of arterial blood flow throughout the kidney, but with limited spatial resolution. Although ASL is not widely employed in the clinical setting, it is expected that this method will gain in importance [75]. For assessment of renal oxygenation, blood oxygenation level–dependent (BOLD) MRI can be used. It is currently the only clinically relevant noninvasive method for assessing renal oxygenation status. The BOLD signal is not only oxygen-dependent, but also influenced by factors such as hydration level, hematocrit, and pH value. Therefore, validated protocols for acquisition and data analysis must be strictly followed [76]. Oxygenation can also be measured with fluorine-19 MRI [77]. This method is employed in pre-clinical studies, requiring the application of perfluorinated hydrocarbons as contrast agents.

Doppler ultrasound is a widely available, inexpensive method for measuring blood velocity with high temporal resolution. However, it has two important shortcomings: Firstly, the measured velocity is dependent on the angle of insonation, which can neither be precisely controlled nor measured, as ultrasound transducers are generally hand-held and not registered to a reference coordinate frame. Secondly, the calculation of blood flow rate from the measured velocities is error prone, as it is generally based on the assumption of exclusively parallel flow along the longitudinal axis of the vessel. Consequently, traditional Doppler ultrasound measurements offer relatively low accuracy outside idealized settings [78]. Newer methods [79], e.g., ones based on a combination of high frame rate and speckle decorrelation [80], promise more reliable estimation of blood flow, but they have yet to be established in the clinic.

### Methods for invasive measurements

A reliable and easy-to-use method for measuring total RBF is transit time ultrasound via intraoperative application or chronic implantation of an ultrasound probe [81]. For intraoperative measurements, electromagnetic probes can be employed as well. They require close contact with the blood vessel, which excludes chronic implantation due to possible vessel wall damage [82]. Microcirculatory perfusion can be assessed by laser Doppler flowmetry using fiber optic probes. However, the obtained signal only provides a relative measure of perfusion, as calibration is tissue-dependent [83]. Dilution indicator and microsphere methods for blood flow measurement have been largely displaced [84].

Renal oxygenation can be assessed with electrochemical sensors [85], luminescence-based optical sensors [86], intravital fluorescence and phosphorescence lifetime microscopy [87], photoacoustic microscopy [88], and electron paramagnetic resonance spectroscopy [89]. Of these, electrochemical and luminescence sensors are the most widely used in the clinical setting. The best-known electrochemical oxygen sensor is the Clark electrode. Formerly considered the reference standard, it is prone to drift and has limited accuracy at low pO_2_ due to oxygen consumption as the basis of its operating principle. Luminescence sensors do not show these issues. However, they have a larger sampling volume and are bigger than Clark microelectrodes, which may lead to more tissue damage [90]. Electron paramagnetic resonance spectroscopy requires the placement of a paramagnetic material in the kidney at the location of intended pO_2_ measurement. The actual measurements are noninvasive, allowing for longitudinal observations [89]. Intravital fluorescence and phosphorescence microscopy requires the use of oxygen-sensitive molecular probes. It yields spatially resolved data, but has lower accuracy and is less robust than electrochemical and luminescence sensor-based methods. Using fiber optic systems, fluorescence imaging is also possible in deep tissue [91].

### Computational approaches

Where measurements do not suffice, mathematical or computational approaches based on first principles, i.e., on fundamental laws of physics, may fill some of the gaps. For instance, on the simplest level, the law of mass conservation dictates that the overall mass of blood supplied to the kidney has to be equal to the mass exiting the kidney as venous blood, urine, and lymph. Therefore, if arterial inflow and venous and urinary outflows can be measured, lymphatic outflow can be calculated. On a more complex level, global or coarse local measurements of RBF and renal tissue oxygenation can be used in conjunction with quantitative data on the renal anatomy to infer local perfusion and oxygen delivery values. Models of biochemical reactions and biophysical actions of, e.g., epithelial membrane transporters can be employed to estimate the local oxygen consumption rate. Combining both types of models allows, in principle, for the calculation of pO_2_ values throughout the kidney.

For an overview of modeling of renal oxygenation, including a general introduction to the application of computational models in renal pathophysiology, we refer the reader to the recent review of Evans and co-workers [92]. Here, we give two examples of how computational models have helped fill in gaps not accessible by measurements. The first one pertains to the question of whether preglomerular arterial-to-venous oxygen shunting plays a role in the oxygenation of renal tissue. This question cannot be answered by direct measurement, as it would require acquisition of oxygen fluxes along the full length of pairs of arterial and venous vessels. In a series of computational models with increasing complexity [93–96], the conclusion was reached that preglomerular oxygen shunting is negligible under normal physiologic conditions, but may have to be accounted for in specific situations, e.g., during ischemia [97]. The second example addresses the question of how enough oxygen can reach the papilla given the countercurrent architecture. A model representing the detailed three-dimensional structural organization of the outer medulla suggested that the segregation of vasa recta within vascular bundles limits O_2_ escape from descending vasa recta in the inner stripe and helps to preserve O_2_ delivery to the inner medulla [98, 99]**.**

The main limitations of current computational models of renal blood flow and oxygenation are related to the multiple length and time scales of kinetic, transport and regulation processes, the sparsity of available quantitative data to characterize these processes, the complex spatial architecture of the kidney, and the high computational power needed to run three-dimensional, time-resolved, multiscale models. To capture time-dependent processes, the equations describing the modeled system are commonly solved in a stepwise fashion, where each step moves the model forward in time, and requires a quantum of computer power. Processes at the cellular level tend to be fast, requiring small time steps to be captured adequately. At organ scale, process cycles tend to be longer and characteristic time constants larger, allowing for the use of bigger time steps. When both scales are modeled concurrently, the computational power needed to compute long cycles with small time steps can exceed the available resources by orders of magnitude. For a discussion of further challenges of and progress in multi-scale modeling, we refer to reader to the review by Auffray and co-workers [100].

Adequately representing the complex organization of vessels and tubules also remains challenging. In addition to the intricate vascular geometry described above, recent studies based on optical clearing methods have revealed great variability in the position, size, shape, and tortuosity of nephrons [101]. Representing each individual structure is prohibitively expensive from a computational perspective, but selecting representative populations that preserve the key features of this anatomical arrangement is difficult, particularly given that the functional significance of this variability remains to be fully understood.

Added to this structural complexity is the complexity of biological processes that determine the local pO_2_ at any given moment in time. A complete model of renal oxygenation may need to accurately represent O_2_ dissociation from hemoglobin, which depends on the acid-base environment, as well as O_2_ diffusion across multiple barriers, the permeabilities of which remain poorly characterized. It should also take into account the efficiency of O_2_ utilization by mitochondria together with ATP-consuming processes other than active transport, all of which are strongly modulated by hormones, neural signals, and local factors. Finally, it should also account for physiologic differences due to sex as well as age-induced changes.

The high number of structures and processes involved in kidney function raise the question whether machine learning approaches such as deep neural networks might not be better suited for modeling renal blood flow and oxygenation than conventional methods. The two main problems associated with machine learning approaches are that they require large amounts of data for training purposes and that they reproduce, rather than provide, functional insight into the modeled system. A new class of neural networks—physics [102], biology [103], or otherwise “informed” neural networks—seeks to merge conventional methods for physiological modeling with machine learning. It is too early to say what impact it will have on kidney research.

## Conclusion

Renal function is both dependent on and the cause of regional variations in renal blood flow and oxygenation. A multitude of interactions between neural, endocrine, paracrine, and autocrine factors work in concert to regulate RBF and pO_2_. To advance our understanding of the interplay between RBF, tissue oxygen, and renal function, spatially resolved measurements are necessary. Currently available measurement methods have limitations in that they introduce confounding factors, have low resolution, or cannot reach deep tissue. Major breakthroughs in this area will likely require hybrid approaches with both experimental methods and computational modeling tools.
